# LipiSensors: Exploiting Lipid Nanoemulsions to Fabricate Ionophore-Based Nanosensors

**DOI:** 10.3390/bios10090120

**Published:** 2020-09-10

**Authors:** Alexandra L. Dailey, Meredith D. Greer, Tyler Z. Sodia, Megan P. Jewell, Tabitha A. Kalin, Kevin J. Cash

**Affiliations:** 1 Chemical and Biological Engineering, Colorado School of Mines, Golden, CO 80401, USA; alexandradailey@alumni.mines.edu (A.L.D.); meredithgreer@alumni.mines.edu (M.D.G.); megan.jewell@colorado.edu (M.P.J.); tkalin@alumni.mines.edu (T.A.K.); 2 Quantitative Biosciences and Engineering, Colorado School of Mines, Golden, CO 80401, USA.; tsodia@mines.edu

**Keywords:** probes, fluorescence, chemosensor, nanoparticle, calcium, oxygen sensing

## Abstract

Ionophore-based nanosensors (IBNS) are tools that enable quantification of analytes in complex chemical and biological systems. IBNS methodology is adopted from that of bulk optodes where an ion exchange event is converted to a change in optical output. While valuable, an important aspect for application is the ability to intentionally tune their size with simple approaches, and ensure that they contain compounds safe for application. Lipidots are a platform of size tunable lipid nanoemulsions with a hydrophobic lipid core typically used for imaging and drug delivery. Here, we present LipiSensors as size tunable IBNS by exploiting the Lipidot model as a hydrophobic structural support for the sensing moieties that are traditionally encased in plasticized PVC nanoparticles. The LipiSensors we demonstrate here are sensitive and selective for calcium, reversible, and have a lifetime of approximately one week. By changing the calcium sensing components inside the hydrophobic core of the LipiSensors to those sensitive for oxygen, they are also able to be used as ratiometric O_2_ sensitive nanosensors via a quenching-based mechanism. LipiSensors provide a versatile, general platform nanosensing with the ability to directly tune the size of the sensors while including biocompatible materials as the structural support by merging sensing approaches with the Lipidot platform.

## 1. Introduction

The ability to quantitatively analyze the fluctuations of ions, metabolites and other relevant molecules in chemical and biological systems is an essential tool for understanding how these complex systems function. Sensing changes in analyte concentration portrays useful information about the system in question that leads to a higher-level of characterization in medicine, agriculture, environmental science, microbiology and molecular biology [1,2,3,4,5]. Necessary towards this goal, sensors at their simplest have two key components: a recognition element that interacts with the analyte and a transducing element to convert that interaction into a measurable readout—which can come in many forms [6].

A key class of chemical sensors are common small molecule responsive dyes. These dyes (e.g., Fura-2 for calcium [7], or Fluorescein for pH [8], among many others [5]), which combine both the recognition and transduction elements into a single small molecule, have revolutionized science through their ability to quantify analytes in complex settings [9]. Despite their efficacy, this class of sensor has several limitations that prevent adoption in some systems. First, they have limited ability to easily change sensor response characteristics such as the response midpoint (e.g., K_d_ or pK_a_). While this can be achieved with synthesis of novel derivatives [10], it is not easily achievable for an end user outside the synthesis space. Additionally, while the small size of these dyes makes them easy to administer to a sample (and with modification into cells), it also makes them vulnerable to wash steps—making extracellular imaging more challenging [11]. Again, with difficult synthetic approaches, covalently tethering probes in the extracellular space is achievable [12], but at the cost of increased complexity.

A complementary technology to molecular probes, ionophore-based nanosensors (IBNS), can be employed as sensing units, as they are easy to fabricate [13], have a highly tunable response [14,15] and are larger than traditional molecular probes which makes it much easier for in vivo [16,17] and extracellular applications [4,18]. IBNS are typically fabricated from a plasticized polymer phase encased in a polyethylene glycol (PEG) surfactant layer to form nanoparticles that encase the necessary sensing moieties inside the hydrophobic polymer phase. Typically, an ionophore is used for recognition, a lipophilic pH-sensitive Nile blue derivative (chromoionophore) for transduction and a charge balancing additive to control response [19]. This sensing approach is based on polymeric optodes [15] (themselves based on ion-selective electrodes), which can vary based on the transducing element [20,21], structural components [22,23], and the target analyte [15]. For a cation-selective IBNS, like the one presented in this work, the ionophore extracts the analyte from the surrounding aqueous solution into the lipophilic core while simultaneously releasing an equivalent charge to maintain electroneutrality within the core. The released charge comes from the deprotonation of the chromoionophore, which results in alteration of its spectroscopic properties (e.g., fluorescence) and can be measured with standard laboratory equipment. Greater detail on ionophore-based optode and nanosensor mechanisms has been provided in multiple reviews [15,19,21].

The structural components of IBNS can be altered from the traditional plasticized PVC fabrications to fit a specific need without hindering the ion exchange mechanism [22,23,24,25]. For example, Balaconis et al. described biodegradable nanosensors to detect sodium with polycaprolactone polymer and a citric acid ester plasticizer while maintaining IBNS character [22]. Additionally, Xie et al. formulated alternatives to the PVC model by encapsulating the sodium sensing components into a bis(2-ethylhexyl) sebacate (DOS) and Pluronic^TM^ F-127 nanoparticle and again, maintained the expected sensing characteristics [23]. This work further demonstrates that the structural materials do not drastically impact the sensor function (assuming key parameters such as hydrophobicity are similar), and that they can be replaced with alternate structural components to adjust other parameters.

One of the limitations of current polymer-based nanosensors is the challenge of finely tuning their size with formulation-based approaches [26]. Others have demonstrated the ability to change nanosensor size through changing out the key components (e.g., the polymer or plasticizer) in each formulation, resulting in functional sensors over several size ranges [23,27]. In this work, we demonstrate the ability to tune sensor size using the same components but altering the formulation ratio of these components. This approach is based on Lipidots [28], which are size tunable, biocompatible waxy nanoparticles used for in vivo imaging applications such as image-guided tumor surgery [29] and targeted phototherapeutic anti-cancer drug delivery [30,31], which we combine with ionophore-based sensing. Traditionally, Lipidots are dye-loaded lipid nanoemulsions (LNEs) with an oil and wax hydrophobic core lined with phosphatidylcholine and a PEG surfactant layer. The size of Lipidots is easily controlled through varying the formulation, enabling rapid development and optimization of nanoparticle size for distribution, targeting, or retention needs [28,32,33]. Like IBNS and bulk optodes, the matrix components of Lipidots are easily manipulated and can incorporate hydrophobic or amphipathic dyes, effectively making their emission tunable from visible into the near-infrared region of the electromagnetic spectrum [28,34,35,36,37]. While Lipidots and other LNEs have effectively been used for imaging and drug delivering agents, they have yet to be exploited for analyte sensing applications.

This work discusses the development and testing of LipiSensors for monitoring multiple analytes—calcium using ionophore-based recognition and oxygen using a luminescence quenching-based approach. We demonstrate a controllable size variance from 120 nm to 320 nm, based on variations of the surfactant to lipid ratio. We show that Lipidots can be manipulated into a novel nanosensor system, LipiSensors, by changing the functional components that reside in the wax/oil core. These nanosensors can monitor analytes using multiple well-characterized mechanisms.

## 2. Materials and Methods

The polyoxyethylene-40-stearate (Myrj^TM^ 52, POE-stearate, surfactant), soybean oil (oil), sodium tetrakis-[3,5-bis(trifluoromethyl)phenyl]borate (NaBARF; Selectophore), Chromoionophore III (CHIII; Selectophore), magnesium chloride hexahydrate, lithium chloride and acetone were purchased from Sigma-Aldrich (St. Louis, MI, USA). The 2-[4-(2-Hydroxyethyl)- piperazin-1-yl]ethanesulfonic acid (HEPES; Molecular Biology grade), 2-amino-2-hydroxymethylpropane-1,3-diol (Tris; 2 M), sodium chloride, potassium chloride, hydrochloric acid concentrate (HCl; 10N, ACS certified), sodium hydroxide concentrate (NaOH; 10N, ACS certified) and 4-Di-16-ASP (4-(4-(Dihexadecylamino)styryl)-N-Methylpyridinium Iodide) (DiA) were purchased from Fisher-Scientific (Waltham, MA, USA). The 5,10,15,20-Tetrakis(pentafluorophenyl)-21 h, 23 h-porphine, platinum(ii) (PtTFPP) was purchased from Frontier Scientific (Logan, UT, USA). The 1,2-dipalmitoyl-sn-glycero-3-phosphocholine (lipid) was from Avanti Polar Lipids, Inc. (Alabaster, AL, USA). The Ca^+2^ Ionophore II was from Santa Cruz Biotechnology (Santa Cruz, CA, USA). The Suppocire NC^TM^ (wax) was a sample from GATTEFOSSÉ USA. The Micro-Dialysis Hollow Fibers (13kDa cutoff) and the Spectra/Por weighted and magnetic weighted closures (for LipiSensor dialysis) were purchased from Spectrum Laboratories, Repligen (Waltham, MA, USA).

To make the calcium LipiSensors, 4 mg of NaBARF and 0.75 mg of Ca^+2^ Ionophore II were weighed in 2-dram scintillation vial and dissolved in 200 μL of acetone. The acetone was evaporated before the wax, lipid and surfactant was added to the vial. The amount of wax and lipid in the solid pre-mixture was held constant (215.5 mg and 25 mg, respectively) while the surfactant differed among the desired size of the sensor (small—258.8 mg, medium—172.5 mg, large—86.3 mg). The solid pre-mixture was dissolved in 74.2 μL of soybean oil (0.97 g/mL) and 1046 μL of 154 mM NaCl in Millipore water. Then, 0.65 mg of Chromoionophore III (5 mg/mL in 130 μL THF) was added just before sonication (10 × 30 s at 30%) while in a water bath at 70 °C (Branson 450 Probe-tip sonicator). The oxygen sensors fabrication follows a similar procedure, although without addition of calcium sensing components (CHIII, NaBARF, Ca^+2^ Ionophore II). Instead of these components, a solution of 5.1 mg PtTFPP (oxygen responsive) and 0.1 mg DiA (oxygen insensitive) in 200 μL THF was injected into the soybean oil/Millipore mixture just before sonication. The sonicated solutions were dialyzed for 12–24 h in HEPES/6 mM Tris (pH 7.4, like all HEPES/Tris used in this work unless specified otherwise), then diluted in HEPES/Tris at 11.1% sensor volume to total volume and stored at room temperature in the dark. Table S1 outlines the formulation of each size of LipiSensor.

The calibration curves were obtained by loading 100 μL samples of Ca^+2^ LipiSensor and 100 μL of sample solution (2 μM–2M CaCl_2_ diluted in HEPES/Tris) into Nunc MicroWell 96-Well Optical-Bottom Plates (Nalgene Nunc International, Roskilde, Denmark). This yields a final volume of 200 μL and a final concentration of 1 μM–1 M CaCl_2_. Additionally, each row had a well containing 100 μL of 0.1 N NaOH and a well containing 0.1N HCl to obtain the minimum and maximum fluorescence of the sensors, respectively (through fully deprotonating or protonating the chromoionophore directly) as well as a well containing a Ca^+2^ free sample of HEPES/Tris. The selectivity of the LipiSensors was determined by running calibration curves with medium sized sensors against other competing analytes (NaCl, KCl, LiCl, and MgCl_2_). For equipment availability, selectivity was tested on Day 3 of the lifetime analysis. For selectivity analysis, each combination of LipiSensor and competing analyte were obtained in triplicate. The calibration curves were obtained with a Synergy H1 microplate reader with an excitation of 650 nm and emission reading of 680 nm. Selectivity coefficients were calculated by:

log (K_Ca, J_) = log (K_Ca_) − log (K_J_) (1)

where K_Ca_ is the concentration of Ca^+2^ and K_J_ is the concentration of a given interfering analyte, both at their respective response midpoints.

After fabrication, the 11.1% LipiSensor solution was diluted 1:3 in HEPES/Tris to obtain the ζ-potential and dynamic light scattering, which were measured on a Brookhaven ZetaPALS with Particle Solutions software V 2.2 (Brookhaven Instruments Corporation, Holtsville, NY, USA).

To test reversibility, medium-sized LipiSensors were placed into a hollow fiber dialysis tube (MWCO 13 kDa, Spectrum Laboratories) via capillary action and sealed at each end, then secured into a traditional culture dish with superglue and a pipette tip [38]. After the superglue hardened the tube was submerged in HEPES/Tris and the initial reading was taken on a Zeiss LSM780 confocal microscope. The buffer solution was removed, and the tube was washed twice with Millipore water before 2 M CaCl_2_ was added and the second reading was taken. The aforementioned buffer-to-2 M CaCl_2_ cycle was repeated three times. The confocal images were processed in Fiji [39].

The functional sensor lifetime of the LipiSensors was observed by Ca^+2^ response, diameter (by DLS) and ζ-potential (Brookhaven ZetaPALS) changes over time. The sensors used for functional lifetime were medium sized (220 nm diameter). A calibration curve was obtained on days 0, 1, 2, 3, 7, and 14 and the diameter and ζ-potential were obtained on days 0, 7, and 14. The response, selectivity, reversibility, and lifetime data were all fit to four-parameter logistic curves and normalized between either 0.1N HCl (Acid) and 0.1N NaOH (Base) or 0M analyte and Base with GraphPad Prism 8.4.3 software (San Diego, CA, USA). All statistical analysis was also done with GraphPad Prism 8 (One-Way ANOVA).

The oxygen sensors (O_2_NS) were calibrated on an Avantes Avaspec—ULS2048L Starline versatile fiber-optic spectrometer (Apeldoorn, the Netherlands) with a 100 μm slit width. The O_2_NS was placed in an airtight quartz cuvette and bubbled with a controlled blend of compressed air and nitrogen to alter the oxygen content in the sensor solution. The spectra were collected at increasing concentrations of oxygen (0–21%) and a linear regression was performed in GraphPad Prism 8 to obtain a pseudo Stern–Volmer plot with each point representing the quotient of the ratio of PtTFPP intensity (at 650 nm) to DiA intensity (at 585 nm) at 0% oxygen (R_f_^0^) and the ratio of PtTFPP to DiA at increasing concentrations of oxygen (R_f_) (instead of using the intensity ratio of the luminophore at increasing oxygen as in a traditional Stern–Volmer plot).

## 3. Results

### 3.1. Mechanism

**Figure 1:**
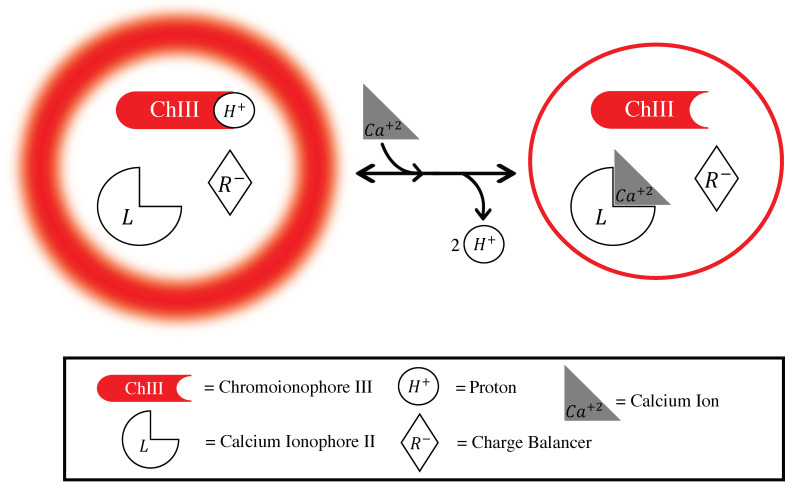
The LipiSensor ion exchange mechanism in response to increasing concentrations of Ca^+2^. The particles have a higher fluorescence at low [Ca^+2^] as the reporter dye (chromoionophore III) is protonated. As [Ca^+2^] increases, it is extracted into the core of the sensor and protons are excluded through ion exchange. This deprotonation is reflected as a decrease in nanosensor fluorescence. This equilibrium-driven process is fully reversible upon decreasing calcium concentration.

We developed LipiSensors based on previous IBNS methodologies combined with the established Lipidot technology [28]. In this work, the Ca^+2^-sensing components consisted of Ca^+2^ ionophore II, Chromoionophore III, and NaBARF (as a charge-balancing additive) with the structural components of Lipidots (a mixture of wax, oil, lipids and POE-stearate surfactant). The mechanism of these nanosensors follows the same ion exchange principles as traditional IBNS and is depicted in Figure 1. At low Ca^+2^ concentrations, the chromoionophore is protonated and has a high fluorescence intensity. As Ca^+2^ concentrations increase, the ionophore binds and extracts the analyte from the aqueous phase into the organic sensor phase, while deprotonation of the chromoionophore simultaneously occurs as a result of ion exchange. This results in a decrease in fluorescence intensity as the chromoionophore deprotonates. Importantly, this is an equilibrium-driven process, and is reversible: as Ca^+2^ concentrations drop, the intensity returns to baseline levels.

### 3.2. Ca^+2^ Response

As described in Lipidot work [28], the size of these nanoparticles can easily be manipulated based on the surfactant-to-lipid ratio. Based on this notion, we fabricated small, medium, and large sensors with diameters of 120 nm, 220 nm, and 320 nm, respectively (Figure S1). The ζ-potential varied between the three different batches with the smallest size LipiSensors having the most negative ζ-potential (−14.8 ± 2.2 mV) and the largest size particles having a smaller magnitude ζ-potential (−6.0 ± 1.3 mV), indicating a difference in surface charge from the three formulations (Figure S1). This highlights that the formulation changes impact both the size and ζ-potential.

**Figure 2:**
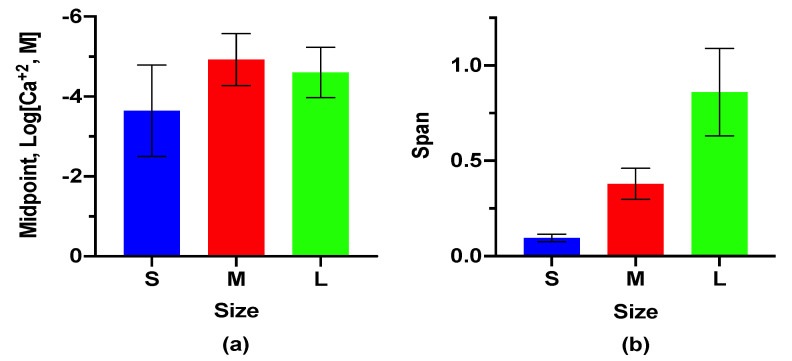
LipiSensors have a clear response to Ca^+2^ (fit from eight measurement points, Figure S2): (**a**) the response midpoint is slightly different (*p* = 0.044) across the three varying sizes; (**b**) the response span (max-min signal) of the LipiSensor varies drastically between the three different sizes (*p* < 0.0001). Error bars depict the 95% CI of the non-linear regression from data in Figure S2.

Each size of the LipiSensor responded to Ca^+2^ in a dose-dependent manner as expected (Figure 2, Figure S2 for calibration curve). The midpoint responses (the concentration where the sensor has responded 50%), as well as their span (the difference in signal between maximum and minimum response), differed from one another, based on size (ANOVA, *p* = 0.044 and *p* < 0.0001, for midpoint and span, respectively). The small sensors had a response midpoint of 230 μM, medium had a midpoint of 12 μM, and large had a midpoint of 25 μM (Figure 2a). These results indicate that the response of the sensors is a function of the sensor matrix formulation, with the span changing significantly, and the response midpoint weakly significantly different. While the results differ based on formulations, the sensor characteristics are highly tunable [14], meaning we could adjust the response parameters with the formulation of the sensing components after selecting an appropriate size formulation.

### 3.3. Lifetime

**Figure 3:**
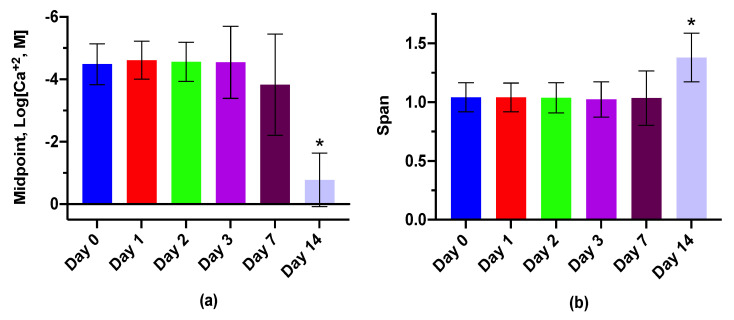
LipiSensors have a functional lifetime of one week. The response character drastically changed between the first and second week (*n* = 3). (**a**) Midpoint response as a function of time. By day 14, the sensors are much less sensitive to Ca^+2^ (* *p* < 0.0001). (**b**) Sensor span as a function of time. Between day 7 and 14, sensor span increases significantly (* *p* = 0.0112).

The medium-sized LipiSensors were used to examine lifetime, selectivity and reversibility. The response character over 14 days post-fabrication is displayed in Figure 3 (calibration curve in Figure S3). The sensors had very similar responses to Ca^+2^ in the first 7 days. By day 14 the sensor character was dramatically impaired relative to earlier days (ANOVA, midpoint *p* < 0.0001, span *p* = 0.0112)—deeming the sensor lifetime of at least three days with similar function on day 7. Furthermore, the DLS displayed an increase in sensor diameter between day 0 and day 7 (236 nm to 260 nm) followed by a decrease of 90 nm by day 14 (to 171 nm). Additionally, the ζ-potential between day 7 and day 14 decreased from −12 mV to −40 mV (Figure S4)—a ζ-potential not typically found in lipid nanoemulsions [32,33]. These changes are likely due to an unknown degradation pathway of the sensors.

### 3.4. Selectivity

**Figure 4:**
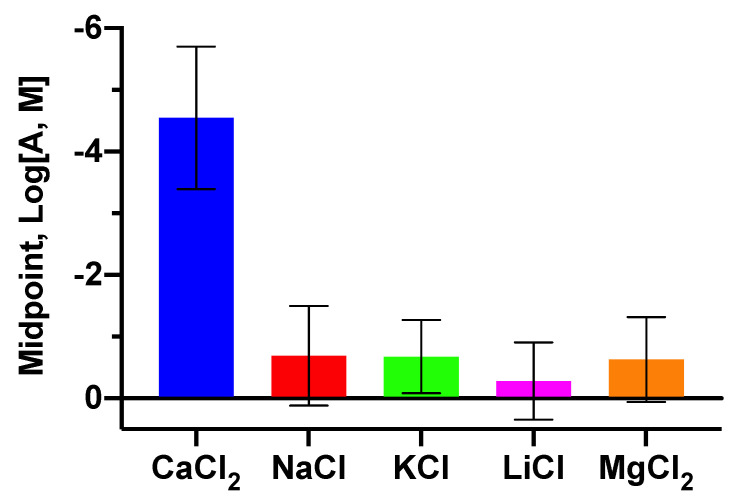
LipiSensors are selective for measuring Ca^+2^ over other analytes (*n* = 3). Midpoint response versus potentially competing ions.

The design of sensors like our LipiSensors requires a selective response to Ca^+2^ because the desired future applications may be complex in their ion makeup, which could hinder accurate Ca^+2^ measurements. Thus, in addition to testing Ca^+2^, we also tested Na^+^, K^+^, Li^+^ and Mg^+2^ chloride salts (0–1 M) on day 3 of the lifetime analysis (Figure 4 and Figure S5 for calibration curve). The resulting midpoints yielded selectivity coefficients (in log_10_, M) of at least −3.86 when comparing Ca^+2^ to each of the competing analytes (K_Ca,Na_ = −3.86, K_Ca,K_ = −3.87, K_Ca,Li_ = −4.26, K_Ca,Mg_ = −3.91). In other words, the competing analytes did not interfere with the LipiSensor response to Ca^+2^ due a selectivity difference of almost four orders of magnitude.

### 3.5. Reversibility

**Figure 5:**
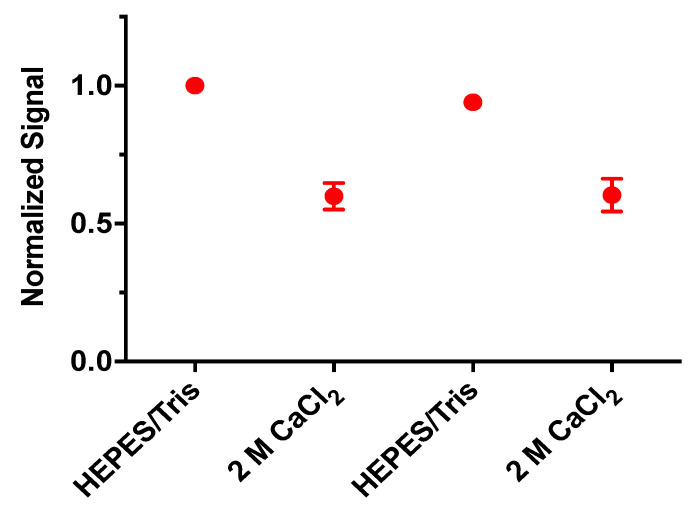
The LipiSensors respond reversibly to changes in [Ca^+2^] (*n* = 3).

The mechanism of IBNS is an equilibrium-based measurement [19]. This means that changes in analyte concentrations will result in a continuous change in fluorescence output while the sensors maintain equilibrium between the aqueous and sensing phase. To determine reversibility, we cannot use the approaches common with larger sensors (e.g., electrochemical sensors), as separation of the nanosensors from the solution is more challenging. As an alternative, we encapsulated nanosensors inside hollow fiber dialysis tubes, which have a molecular weight cutoff of 13 kDa. This means the tubes retain the nanosensors, while being permeable to Ca^+2^ and competing analytes. This approach allows for dynamic imaging and measurements while altering the concentration of Ca^+2^ in the surrounding buffer solution. Cycling the Ca^+2^ concentration in the solution surrounding the dialysis tube resulted in the expected response and reversibility in fluorescence signal of the LipiSensors (Figure 5).

### 3.6. Oxygen-Sensitive LipiSensor Response and Characterization

**Figure 6:**
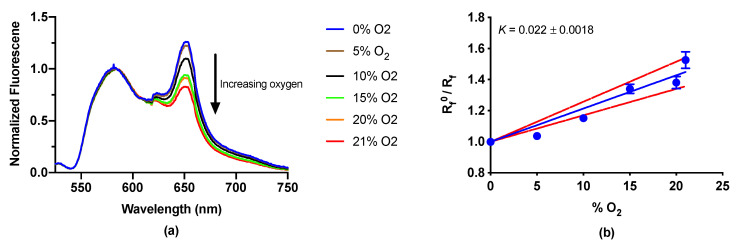
LipiSensors are effective at sensing oxygen. (**a**) Normalized spectrum of Lipisensors with varying oxygen concentrations (normalized to intensity at 585 nm, emission maximum of DiA). (**b**) Pseudo Stern–Volmer plot showing that oxygen quenches the luminescence of PtTFPP as expected (*n* = 3). Red dashed lines depict a 95% confidence interval on the fit.

To expand on the functionality of the LipiSensor design, we also developed LipiSensors for sensing oxygen concentration based on collisional quenching of a metal centered porphyrin dye, a commonly used approach in optical oxygen sensors and reviewed extensively in [40,41,42]. Here, we used the medium-sized LipiSensor formulation with Platinum tetra-fluorophenyl porphyrin (PtTFPP) as the oxygen-responsive dye and DiA as an oxygen-insensitive reference dye to enable ratiometric measurements—as we have done before in oxygen sensing [4,18]. When oxygen concentrations increase, the fluorescence of the PtTFPP at 650 nm decreases through quenching while the DiA at 585 nm is unaffected (Figure 6a). Dissolved oxygen was measured between 0 and 21%, showing the expected increase in quenching with increasing oxygen, and a measured ratiometric quenching constant of 0.022 (Figure 6b).

## 4. Discussion

Waxy lipid nanoparticles known as Lipidots can serve as the structural backbone for selective optical nanosensors. We used the well-characterized sensing mechanism of IBNS coupled with the structural composition of Lipidots for size tunability [28]. As our LipiSensors are Lipidots with Ca^+2^- or oxygen-sensing capability, the diameter was easily tunable with changes in the mass ratio of surfactant to lipid (~10 for 120 nm, ~7 for 220 nm, ~3.5 for 320 nm). More detail on lipid nanoemulsion-based size control and stability is discussed in [32,33].

Size control of IBNS presents the opportunity to expand and optimize the size-dependent functionality of the sensors. Our typical IBNS are ~200 nm in diameter [20], with minimal tunability to the size. Larger sensors can be used for better entrapment in microenvironments such as biofilms and other extracellular spaces to better understand the spatiotemporal gradients of Ca^+2^ among neighboring cells, which could lead to a deeper understanding of Ca^+2^ signaling [43]. Furthermore, larger sensors have been shown to have less mobility in applications that require stagnancy [44].

The large sensors are still less than half a micrometer in diameter with the reported formulations. However, larger sizes of LipiSensor may be explored with further manipulation, such as a shorter sonication time, variation of surfactant molecular weight, or a decreased ratio of surfactant to lipid [28,45]. Exploring these possibilities may result in alteration of particle stability; however, stability issues with Lipidots and other LNE models has been combated previously by entropic stabilization [32]. While large LipiSensors can be used for extracellular and in vivo applications, the small sensors can be used for small intracellular compartments to detect the compartmentalization of Ca^+2^, transmembrane Ca^+2^ dynamics, and potentially aiding in characterization of Ca^+2^ flux from organelles to the cytoplasmic space (and vice versa) [43,46,47]. The small LipiSensors in this work are ~120 nm in diameter, but it may be possible to formulate much smaller sizes, as other groups have shown Lipidots with 25 nm [28] and IBNS with 20 nm diameters [27].

While size variation is easily achievable, the magnitude of response to Ca^+2^ between each size variant is different (ANOVA, *p* < 0.0001) (Figure 2). Each sensor has a dose-dependent response which is only slightly different from the other sizes as the *p*-value approaches statistical overlap (ANOVA, *p* = 0.044). Conversely, the protonation state of the chromoionophore at zero analyte (measured by span) is varying significantly among the size of sensor (Figure S2). This is likely a result of the formulation change between sizes, where varying the amount of surfactant in the formulation with the goal of changing particle size also shifts the ratio of the charged lipid component to the sensing components. While the notable sensing characteristics (sensitivity, selectivity, dynamic range, reversibility, etc.) are generally a product solely of the sensing components inside the lipophilic core for optode sensors, the observed divergence in fluorescence at zero analyte indicates that the charged lipid component impacts the charge balance, altering the protonation state across the different sizes of sensor. Importantly, this change is addressable for a given application through altering the ratio of the charge balancing additive for a given size-based formulation.

Despite the novel matrix of our sensors, they are functional because of the character that the matrix provides. The matrix of IBNS serve two primary roles: a hydrophobic environment and structural support. The LipiSensor model provides both of these attributes to the sensors, while additionally providing the ability to alter the particle size. Importantly, this structural support function should also work with other materials while maintaining the sensing mechanism as long as the material properties are similar. The fundamental mechanism of IBNS has been demonstrated in a wide range of supporting matrices and is not impacted [22,23,24,25], as others have demonstrated that it is possible to make sensors with only plasticizer [24], brush block copolymers [27], surfactant alone [23], and even SiO_2_ nanoparticles [25].

Traditional bulk optode sensors are limited by low surface area-to-volume ratios, which results in slow mass transport and therefore slow response times. Response time at the nanosensor scale is not a concern. Transitioning to IBNS from bulk optodes increases the surface to volume ratio which allows for faster analyte transport and thus, much quicker response times [19]. Most IBNS, LipiSensors included, respond nearly instantaneously [19].

These sensors have all of the expected characteristics of ionophore-based and oxygen-sensitive nanosensors—including sensitivity for the target, selectivity over competing analytes, reversibility, and a lifetime of at least three days, with similar response at one week. For that reason, they are expandable to other ion sensors as this approach is flexible and tunable due to the separation of the recognition element and optical reporter [14,15]. The oxygen sensors are also expandable to small molecule targets and metabolites through combination with oxidase enzymes—as shown with histamine and glucose [38,48].

LipiSensors are suitable for future in vivo and implantable applications. It is important to note that the structural components of these sensors, adopted from Lipidots, are biocompatible and generally regarded as safe [28,35,37]. Though current formulations of LipiSensors may need to be reformulated to track analytes in a specific physiologically relevant range, they are sensitive and selective (Figure 2 and Figure 4), reversibly responsive (Figure 5), and their adopted Lipidot components have suitable properties for in vivo use [28,34,35,37].

Finally, while in this report we used fluorescence readout, we could have used a wide range of already-demonstrated readouts from other transducing elements for these optode-based sensors, including photoacoustics [49], colorimetry [50], hue determination [51], or even MRI [52], without a change in response character, although some formulation changes will likely be necessary [53].

## 5. Conclusions

In this report, we demonstrated Ca^+2^-sensitive and oxygen-sensitive nanosensors based on Lipidots—lipid nanoemulsions that were originally formulated to function as an in vivo imaging tool. Our LipiSensors have identical mechanisms to previously developed nanosensors, while having the ability to adjust their size for the necessary application (e.g., small for subcellular application, large for extracellular monitoring, implantation and minimal mobility). The future directions for these size-controllable nanosensors are not limited to in vitro diagnostics, as the formulation is biocompatible for in vivo use [28,34,35,37]. The functional lifetime is long enough for many applications, while the selectivity for Ca^+2^ is high and is comparable to other reports [46]. This work provides an approach to incorporate sensing into Lipidots, and a method for enabling size tuning of ionophore-based nanosensors. 

## Supplementary Materials

The following are available online at https://www.mdpi.com/2079-6374/10/9/120/s1, Table S1: Formulations, Figure S1: Diameter and Zeta Potential v. Size, Figure S2: Size and Calibration, Figure S3: Lifetime Calibration, Figure S4: Diameter and Zeta Potential v. Time, Figure S5: Selectivity Calibration, Figure S6: Confocal Images: Reversibility.

## References

[1] Pendley B.D., Lindner E. (2017). Medical Sensors for the Diagnosis and Management of Disease: The Physician Perspective. ACS Sens..

[2] Jett S.E., Bonham A.J. (2017). Reusable Electrochemical DNA Biosensor for the Detection of Waterborne Uranium. ChemElectroChem.

[3] Zhang X.-Y., Yang Y.-S., Wang W., Jiao Q.-C., Zhu H.-L. (2020). Fluorescent Sensors for the Detection of Hydrazine in Environmental and Biological Systems: Recent Advances and Future Prospects. Coord. Chem. Rev..

[4] Jewell M.P., Saccomano S.C., David A.A., Harris J.K., Zemanick E.T., Cash K.J. (2020). Nanodiagnostics to Monitor Biofilm Oxygen Metabolism for Antibiotic Susceptibility Testing. Analyst.

[5] Carter K.P., Young A.M., Palmer A.E. (2014). Fluorescent Sensors for Measuring Metal Ions in Living Systems. Chem. Rev..

[6] Morales M.A., Halpern J.M. (2018). Guide to Selecting a Biorecognition Element for Biosensors. Bioconjug. Chem..

[7] Tsien R.Y. (1980). New Calcium Indicators and Buffers with High Selectivity against Magnesium and Protons: Design, Synthesis, and Properties of Prototype Structures. Biochemistry.

[8] Han J.Y., Burgess K. (2010). Fluorescent Indicators for Intracellular PH. Chem. Rev..

[9] Chan J., Dodani S.C., Chang C.J. (2012). Reaction-Based Small-Molecule Fluorescent Probes for Chemoselective Bioimaging. Nat. Chem..

[10] Qi J., Liu D.Y., Liu X.Y., Guan S.Q., Shi F.L., Chang H.X., He H.R., Yang G.M. (2015). Fluorescent PH Sensors for Broad-Range PH Measurement Based on a Single Fluorophore. Anal. Chem..

[11] Deng F., Liu L., Qiao Q., Huang C., Miao L., Xu Z. (2019). A General Strategy to Develop Cell Membrane Fluorescent Probes with Location- and Target-Specific Fluorogenicities: A Case of a Zn^2+^ Probe with Cellular Selectivity. Chem. Commun..

[12] Hirata T., Terai T., Yamamura H., Shimonishi M., Komatsu T., Hanaoka K., Ueno T., Imaizumi Y., Nagano T., Urano Y. (2016). Protein-Coupled Fluorescent Probe to Visualize Potassium Ion Transition on Cellular Membranes. Anal. Chem..

[13] Dubach J.M., Balaconis M.K., Clark H.A. (2011). Fluorescent Nanoparticles for the Measurement of Ion Concentration in Biological Systems. J. Vis. Exp..

[14] Ferris M.S., Katageri A.G., Gohring G.M., Cash K.J. (2018). A Dual-Indicator Strategy for Controlling the Response of Ionophore-Based Optical Nanosensors. Sens. Actuators B Chem..

[15] Mistlberger G., Crespo G.A., Bakker E. (2014). Ionophore-Based Optical Sensors. Annu. Rev. Anal. Chem..

[16] Ruckh T.T., Clark H.A. (2014). Implantable Nanosensors: Toward Continuous Physiologic Monitoring. Anal. Chem..

[17] Di W., Clark H.A. (2020). Optical Nanosensors for: In Vivo Physiological Chloride Detection for Monitoring Cystic Fibrosis Treatment. Anal. Methods.

[18] Jewell M.P., Galyean A.A., Kirk Harris J., Zemanick E.T., Cash K.J. (2019). Luminescent Nanosensors for Ratiometric Monitoring of Three-Dimensional Oxygen Gradients in Laboratory and Clinical Pseudomonas Aeruginosa Biofilms. Appl. Environ. Microbiol..

[19] Xie X., Bakker E. (2015). Ion Selective Optodes: From the Bulk to the Nanoscale. Anal. Bioanal. Chem..

[20] Galyean A.A., Behr M.R., Cash K.J. (2018). Ionophore-Based Optical Nanosensors Incorporating Hydrophobic Carbon Dots and a PH-Sensitive Quencher Dye for Sodium Detection. Analyst.

[21] Xie X. (2016). Renovating the Chromoionophores and Detection Modes in Carrier-Based Ion-Selective Optical Sensors. Anal. Bioanal. Chem..

[22] Balaconis M.K., Clark H.A. (2012). Biodegradable Optode-Based Nanosensors for in Vivo Monitoring. Anal. Chem..

[23] Xie X., Mistlberger G., Bakker E. (2013). Ultrasmall Fluorescent Ion-Exchanging Nanospheres Containing Selective Ionophores. Anal. Chem..

[24] Ruckh T.T., Mehta A.A., Dubach J.M., Clark H.A. (2013). Polymer-Free Optode Nanosensors for Dynamic, Reversible and Ratiometric Sodium Imaging in the Physiological Range. Sci. Rep..

[25] Du X.F., Yang L.Y., Hu W.C., Wang R.J., Zhai J.Y., Xie X.J. (2018). A Plasticizer-Free Miniaturized Optical Ion Sensing Platform with Ionophores and Silicon-Based Particles. Anal. Chem..

[26] Rao J.P., Geckeler K.E. (2011). Polymer Nanoparticles: Preparation Techniques and Size-Control Parameters. Prog. Polym. Sci..

[27] Du X., Wang R., Zhai J., Li X., Xie X. (2020). Ionophore-Based Ion-Selective Nanosensors from Brush Block Copolymer Nanodots. ACS Appl. Nano Mater..

[28] Gravier J.J., Garcia F.P.N.Y., Delmas T., Mittler F., Couffin A.-C., Vinet F., Texier-Nogues I. (2011). Lipidots: Competitive Organic Alternative to Quantum Dots for in Vivo Fluorescence Imaging. J. Biomed. Opt..

[29] Fortin P.-Y., Genevois C., Koenig A., Heinrich E., Texier-Nogues I., Couillaud F. (2012). Detection of Brain Tumors Using Fluorescence Diffuse Optical Tomography and Nanoparticles as Contrast Agents. J. Biomed. Opt..

[30] Hinger D., Navarro F., Käch A., Thomann J.-S., Mittler F., Couffin A.-C., Maake C. (2016). Photoinduced Effects of M-Tetrahydroxyphenylchlorin Loaded Lipid Nanoemulsions on Multicellular Tumor Spheroids. J. Nanobiotechnol..

[31] Hinger D., Gräfe S., Navarro F., Spingler B., Pandiarajan D., Walt H., Couffin A.-C., Maake C. (2016). Lipid Nanoemulsions and Liposomes Improve Photodynamic Treatment Efficacy and Tolerance in CAL-33 Tumor Bearing Nude Mice. J. Nanobiotechnol..

[32] Delmas T., Piraux H., Couffin A.-C., Texier I., Vinet F., Poulin P., Cates M.E., Bibette J. (2011). How to Prepare and Stabilize Very Small Nanoemulsions. Langmuir.

[33] Delmas T., Couffin A.-C., Bayle P.A., de Crécy F., Neumann E., Vinet F., Bardet M., Bibette J., Texier I. (2011). Preparation and Characterization of Highly Stable Lipid Nanoparticles with Amorphous Core of Tuneable Viscosity. J. Colloid Interface Sci..

[34] Jacquart A., Keramidas M., Vollaire J., Boisgard R., Pottier G., Rustique E., Mittler F., Navarro F., Boutet J., Coll J.-L. (2013). LipImage^TM^ 815: Novel Dye-Loaded Lipid Nanoparticles for Long-Term and Sensitive in Vivo near-Infrared Fluorescence Imaging. J. Biomed. Opt..

[35] Gravier J., Sancey L., Coll J.L., Hirsjärvi S., Benoît J.P., Vinet F., Texier I. FRET as a Tool for the Investigation of the Fate of Lipidots Contrast Agents in Vivo. Proceedings of the SPIE.

[36] Klymchenko A.S., Roger E., Anton N., Anton H., Shulov I., Vermot J., Mely Y., Vandamme T.F. (2012). Highly Lipophilic Fluorescent Dyes in Nano-Emulsions: Towards Bright Non-Leaking Nano-Droplets. RSC Adv..

[37] Mérian J., Boisgard R., Bayle P.-A., Bardet M., Tavitian B., Texier I. (2015). Comparative Biodistribution in Mice of Cyanine Dyes Loaded in Lipid Nanoparticles. Eur. J. Pharm. Biopharm..

[38] Cash K.J., Clark H.A. (2013). Phosphorescent Nanosensors for in Vivo Tracking of Histamine Levels. Anal. Chem..

[39] Schindelin J., Arganda-Carreras I., Frise E., Kaynig V., Longair M., Pietzsch T., Preibisch S., Rueden C., Saalfeld S., Schmid B. (2012). Fiji: An Open-Source Platform for Biological-Image Analysis. Nat. Methods.

[40] Lee S.-K., Okura I. (1997). Photostable Optical Oxygen Sensing Material: Platinum Tetrakis (Pentafluorophenyl) Porphyrin Immobilized in Polystyrene. Anal. Commun..

[41] Papkovsky D.B., Ponomarev G.V., Trettnak W., Oleary P. (1995). Phosphorescent Complexes of Porphyrin Ketones: Optical Properties and Application to Oxygen Sensing. Anal. Chem..

[42] Quaranta M., Borisov S.M., Klimant I. (2012). Indicators for Optical Oxygen Sensors. Bioanal. Rev..

[43] Clapham D.E. (2007). Calcium Signaling. Cell.

[44] Ozaydin-Ince G., Dubach J.M., Gleason K.K., Clark H.A. (2011). Microworm Optode Sensors Limit Particle Diffusion to Enable in Vivo Measurements. Proc. Natl. Acad. Sci. USA.

[45] Hirsjärvi S., Dufort S., Gravier J., Texier I., Yan Q., Bibette J., Sancey L., Josserand V., Passirani C., Benoit J.-P. (2013). Influence of Size, Surface Coating and Fine Chemical Composition on the in Vitro Reactivity and in Vivo Biodistribution of Lipid Nanocapsules versus Lipid Nanoemulsions in Cancer Models. Nanomed. Nanotechnol. Biol. Med..

[46] Rong G., Kim E.H., Poskanzer K.E., Clark H.A. (2017). A Method for Estimating Intracellular Ion Concentration Using Optical Nanosensors and Ratiometric Imaging. Sci. Rep..

[47] Dubach J.M., Das S., Rosenzweig A., Clark H.A. (2009). Visualizing Sodium Dynamics in Isolated Cardiomyocytes Using Fluorescent Nanosensors. Proc. Natl. Acad. Sci. USA.

[48] Ferris M.S., Elms M.K., Cash K.J. (2019). Enzyme-Conjugated Nanosensors with Tunable Detection Limits for Small Biomolecule Determination. AIChE J..

[49] Cash K.J., Li C., Xia J., Wang L.V., Clark H.A. (2015). Optical Drug Monitoring: Photoacoustic Imaging of Nanosensors to Monitor Therapeutic Lithium in Vivo. ACS Nano.

[50] Suzuki K., Hirayama E., Sugiyama T., Yasuda K., Okabe H., Citterio D. (2002). Ionophore-Based Lithium Ion Film Optode Realizing Multiple Color Variations Utilizing Digital Color Analysis. Anal. Chem..

[51] Erenas M.M., Cantrell K., Ballesta-Claver J., de Orbe-Payá I., Capitán-Vallvey L.F. (2012). Use of Digital Reflection Devices for Measurement Using Hue-Based Optical Sensors. Sens. Actuators B Chem..

[52] Chan K.W.Y., Liu G., Song X., Kim H., Yu T., Arifin D.R., Gilad A.A., Hanes J., Walczak P., van Zijl P.C.M. (2013). MRI-Detectable pH Nanosensors Incorporated into Hydrogels for In Vivo Sensing of Transplanted-Cell Viability. Nat. Mater..

[53] Rong G., Tuttle E.E., Neal Reilly A., Clark H.A. (2019). Recent Developments in Nanosensors for Imaging Applications in Biological Systems. Annu. Rev. Anal. Chem..

