# Supplementary material for: LipiSensors: Exploiting Lipid Nanoemulsions to Fabricate Ionophore-Based Nanosensors
Source: Biosensors (Basel). 2020 Sep 10;10(9):120. doi: 10.3390/bios10090120 (PMC7557773; doi:10.3390/bios10090120)
Supplement: Supplementary file 1 [file biosensors-10-00120-s001.pdf]

Supplemental Information For

LipiSensors: Exploiting Lipid Nanoemulsions to Fabricate Ionophore Based Nanosensors

Alexandra L. Dailey [1], Meredith D. Greer [1], Tyler Z. Sodia [2], Megan P. Jewell [1], Tabitha A. Kalin [1] and Kevin J. Cash [1,2,*]

[1] Chemical and Biological Engineering, Colorado School of Mines, Golden, 80401 CO, USA; alexandradailey@alumni.mines.edu (A.L.D.); meredithgreer@alumni.mines.edu (M.D.G.); megan.jewell@colorado.edu (M.P.J.); tkalin@alumni.mines.edu (T.A.K.)

[2] Quantitative Biosciences and Engineering, Colorado School of Mines, Golden, 80401 CO, USA.; tsodia@mines.edu

* Correspondence: kcash@mines.edu; Tel.: +1-303-273-3631

Received: 19 August 2020; Accepted: 8 September 2020; Published: date

Table S1. LipiSensor formulations and their respective diameter. 1: polyoxyethylene-40-stearate. 2: 1,2-dipalmitoyl-sn-glycero-3-phosphocholine. 3: Suppocire NC™. 4: soybean oil.

	Surfactant[1] (mg)	Lipid[2] (mg)	Wax[3] (mg)	Oil[4] (μL)	Ca^{+2} Ionophore (mg)	NaBARF (mg)	CHIII (mg)	Saline (μL)	Approximate Diameter (nm)	DLS Diameter (nm)
Small	258.8	25	215.5	74.2	3.75	6.0	0.65	1046	120	142 ± 32.7
Medium	172.5	25	215.5	74.2	3.75	6.0	0.65	1046	220	226 ± 5.71
Large	86.3	25	215.5	74.2	3.75	6.0	0.65	1046	320	322 ± 8.06

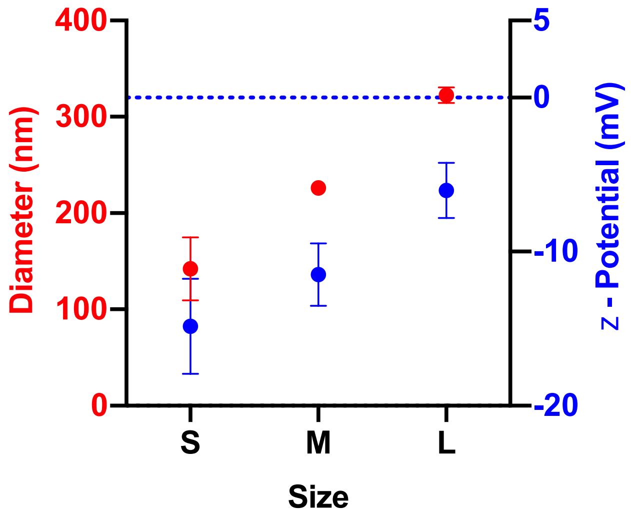

Figure S1. Changing the LipiSensor formulation changes both the size and ζ -potential of the particles. As size increases, the ζ -potential becomes less negative.

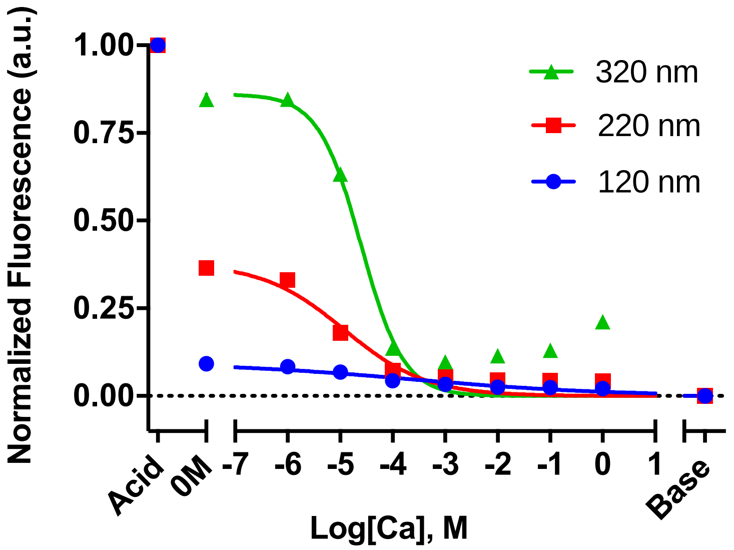

Figure S2. LipiSensors of each size have a dose-dependent response to increasing [Ca^{+2}]. Each size has slightly different response character (see Figure 2).

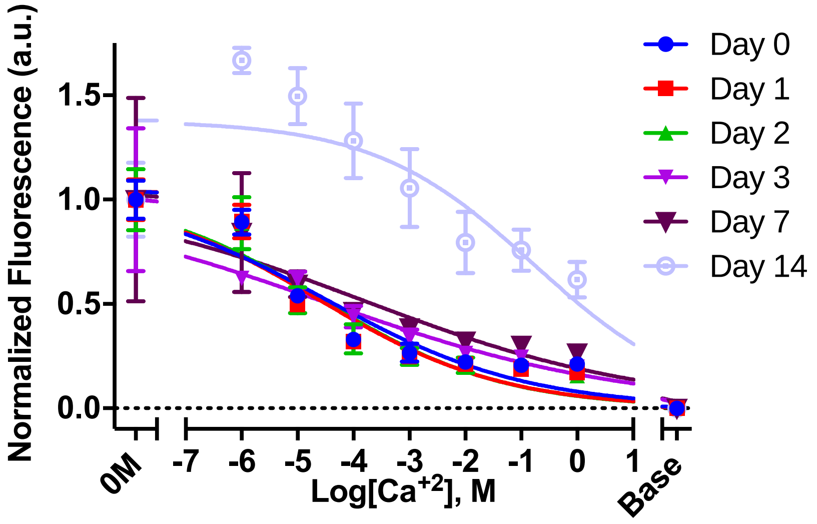

Figure S3. Between day 7 and day 14 the sensor response drastically changes, likely as a result of sensor degradation.

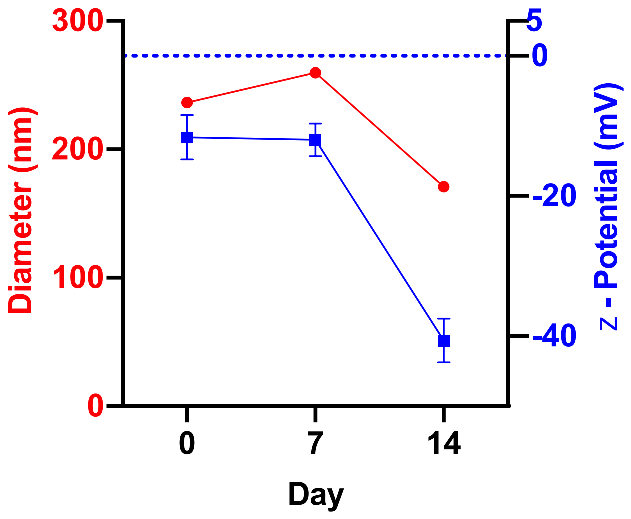

Figure S4. Between day 7 and day 14 the size and ζ -potential of the LipiSensors (medium) are impacted dramatically, also likely as a result of sensor degradation.

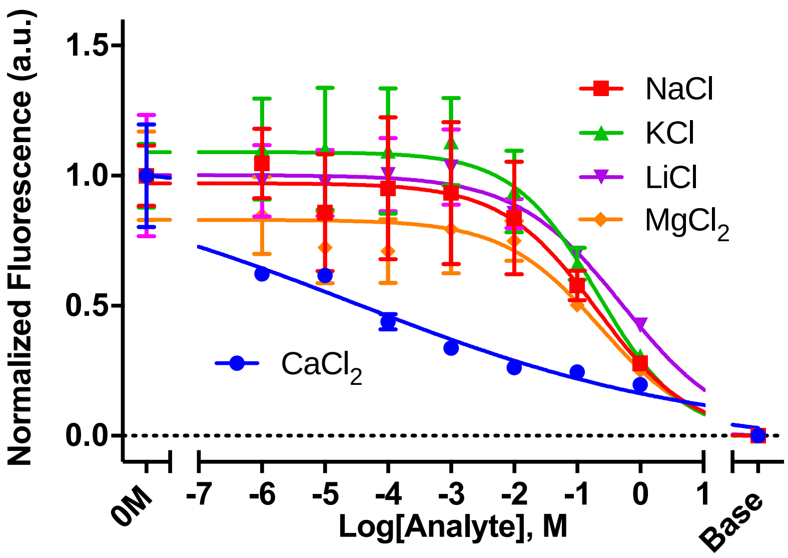

Figure S5. LipiSensors have a selective response to Ca^{+2} (n = 3).

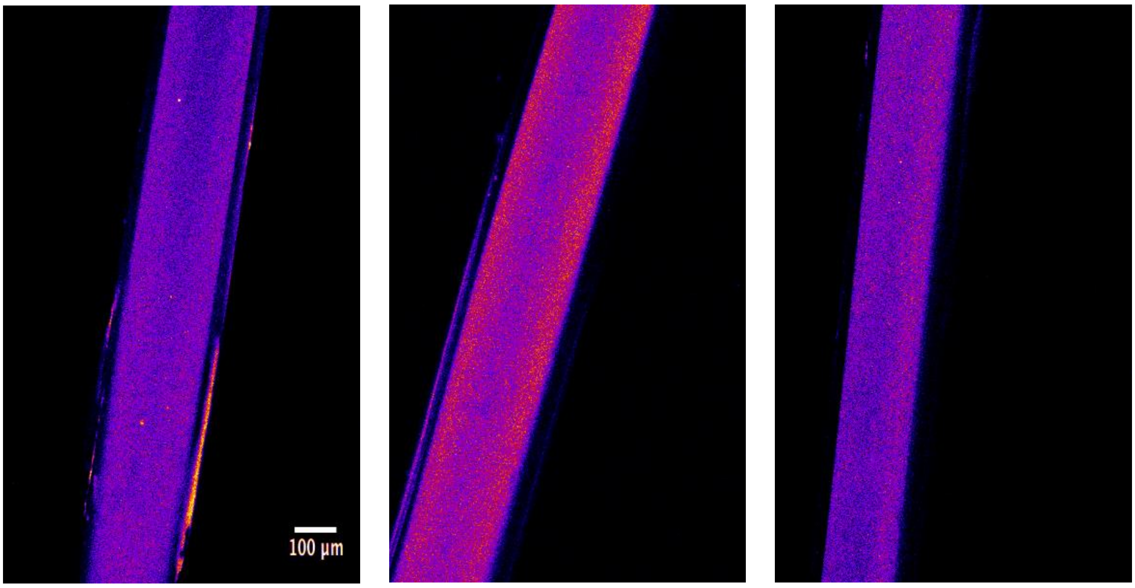

2 M $CaCl_2$ | **HEPES/Tris** | **2 M $CaCl_2$**

Figure S6. LipiSensors have a reversible response to changing [Ca^{+2}]. Dialysis tube (13kDa cutoff) with LipiSensors entrapped inside. Images taken with LSM-780 Confocal Microscope.
